# Hypoxia changes chemotaxis behaviour of mesenchymal stem cells via HIF‐1α signalling

**DOI:** 10.1111/jcmm.14091

**Published:** 2019-01-09

**Authors:** Wei Xu, Ruijun Xu, Zhikun Li, Yi Wang, Ruixi Hu

**Affiliations:** ^1^ Department of Orthopedic Surgery, TongRen Hospital, School of Medicine Shanghai JiaoTong University Shanghai China

**Keywords:** chemotaxis, HIF‐1α signalling, hypoxia, mesenchymal stem cells

## Abstract

Mesenchymal stem cells (MSCs) have drawn great attention because of their therapeutic potential. It has been suggested that intra‐venous infused MSCs could migrate the site of injury to help repair the damaged tissue. However, the mechanism for MSC migration is still not clear so far. In this study, we reported that hypoxia increased chemotaxis migration of MSCs. At 4 and 6 hours after culturing in hypoxic (1% oxygen) conditions, the number of migrated MSCs was significantly increased. Meanwhile, hypoxia also increased the expression of HIF‐1α and SDF‐1. Using small interference RNA, we knocked down the expression of HIF‐1α in MSCs to study the role of HIF‐1α in hypoxia induced migration. Our data indicated that knocking down the expression of HIF‐1α not only abolished the migration of MSCs, but also reduced the expression of SDF‐1. Combining the results of migration assay and expression at RNA and protein level, we demonstrated a novel mechanism that controls the increase of MSCs migration. This mechanism involved HIF‐1α mediated SDF‐1 expression. These findings provide new insight into the role of HIF‐1α in the hypoxia induced MSC migration and can be a benefit for the development of MSC‐based therapeutics for wound healing.

## 1 INTRODUCTION

The bone marrow (BM) stroma does not just contain a heterogeneous population of structural cells, including endothelial cells, fibroblasts, adipocytes, osteogenic cells, but also harbours the niche of stem cells like haematopoietic stem cells and mesenchymal stem cells.^1^ Mesenchymal stem cells have the potential to protect, repair and possibly regenerate the damaged tissue upon arriving the injury site. Bone marrow‐derived mesenchymal stem cells (MSCs) are a promising cell source for such cellular therapy because of their ability for self‐renewal, multipotency and immunosuppressive properties.^2^ Bone marrow MSCs are commonly thought to reside in hypoxic niches in the bone marrow, important for maintaining their undifferentiated state.^3^ This suggests that oxygen tension plays an important role in stem cell regulation and indeed oxygen tension was recently found to affect MSC differentiation.^4^ However, it is not clear if the migration of MSCs is also influenced by oxygen tension.

Mesenchymal stem cells are known as suitable cells that secrete several anti‐inflammatory, angiogenic and antifibrotic factors and induce immunomodulation without significant activation of the immune response. Thus, MSCs induce regeneration in the surrounding tissues and cells, which is an important beneficial effect in cell therapy.^5^ The systemic administration of stem cells is generally preferred to local injection due to being less invasive.^6^ One critical barrier to effective MSC therapy is their insufficient homing capability to tissues of interest, especially when MSCs are infused through the vascular route.^7^ Many factors contribute to the inefficient migration of these cells, among which low surface receptors’ level is of high importance.^8^ Some studies have highlighted that chemokine/‐chemokine receptor interactions influence stem cell recruitment to the desired target, but to the best of our knowledge none of them have investigated whether hypoxia improves the migration of MSCs.

Wound healing is a complex process requiring cell migration, inflammation, angiogenesis, granulation tissue formation, re‐epithelialization and extracellular matrix (ECM) remodelling.^9^ MSCs have an active role through this process, and therapeutic application of MSCs has been shown to enhance and improve wound‐healing outcomes.^10^ Understanding the mechanism of MSCs migration upon chemotaxis stimuli would benefit the development of novel methods that can be used to increase MSC delivery and efficacy for treating wound healing.

In this study, we conducted investigations on the chemotaxis migration of MSCs under hypoxic condition. We also demonstrated the possible mechanism of the MSCs migration at molecular level.

## 2 MATERIALS AND METHODS

### 2.1 Isolation of bone marrow mesenchymal stem cells

Male inbred BALB/C mice, C57BL/6J mice (8 weeks old) were purchased from XXX. All experiments were performed after the approval by our local ethical committee at XXXX. The MSCs were isolated and cultured using standard protocols. Bone marrow cells from C57BL/6 mice were harvested by flushing the femurs and tibias. Then cells were seeded in DMEM with high glucose (Gibco, Grand Island, NY, USA), supplemented with 10% FBS (Lonza, Allendale, NJ, USA), 100 U/mL penicillin and 100 µg/mL streptomycin (Gibco; Thermo Fisher Scientific, Inc, Waltham, MA, USA). The medium was slowly changed after 3 days to remove non‐adherent cells. Meantime, the adherent marrow cells were subcultured until obtaining purify population of mesenchymal stem cells with spindle‐shaped morphology.^11^, ^12^, ^13^

### 2.2 Cell culture and hypoxic conditions

Monolayer cultures of MSCs were routinely maintained in DMEM supplemented with 10% fetal bovine serum (Lonza), 100 U/mL penicillin and 100 µg/mL streptomycin (Gibco; Thermo Fisher Scientific, Inc), at 37ºC in a humidified atmosphere of 95% air and 5% CO_2_. Normoxia was considered as 21% O_2_ (ppO_2_ 588 mm Hg). Hypoxia was generated by a pre‐equilibrated Bactrox hypoxic chamber (Shel Lab; Sheldon Manufacturing, Inc, Cornelius, OR, USA) and oxygen was balanced with N_2_ and CO_2_. Once 90% confluence was reached, MSCs were incubated for 24 hours under moderate (1% O_2_).

### 2.3 Characterization of bone marrow mesenchymal stem cells

Cells were harvested at the fourth passage. After three washes with phosphate buffered saline (PBS) with 1% FBS, the cells were incubated in the dark for 25 minutes with a fluorochrome‐conjugated primary antibodies against CD44 (cat no. 11‐0441‐82; Thermo‐Fisher Scientific, Grand Island, NY, USA), CD73 (cat no. 11‐0739‐42; Thermo‐Fisher Scientific), Sca‐1 (cat no. 550741; BD Biosciences, La Jolla, CA, USA) and CD45 (cat no. 559864; BD Biosciences). Followed by washing with PBS, samples were analysed on a BD LSR II (BD Bioscience).

### 2.4 In vitro migration assays

Migration assays were performed in 96 well transwells with 8 μm pore size filters (Costar™; Corning, NY, USA). In MSC chemotaxis assays, 2.5 × 10^4^ cells were seeded in upper chamber and cultured in serum‐free medium for 24 hours. Cells were then allowed to migrate towards lower chamber for overnight. Migrating cells were stained with methylene blue and counted in four randomly chosen fields (10×) under microscope.

### 2.5 RNA interference and transient infection

For the design of effective siRNA HIF‐1α target sequences, a siRNA design tool was applied siRNA design tool (eurofins, USA) and siRNA target sequences were obtained according to published criteria.^14^, ^15^ For synthesis of siRNA HIF‐1α via in vitro transcription, the Silencer™ siRNA Construction Kit (Ambion, Austin, TX, USA) was used with modifications.^16^ Chemically synthesized GFP siRNA HIF‐1α was bought from Ambion (Ambion). For transient infection, cells were cultured and grown to 70%‐90% confluence, and then transfected with siRNAs using Lipofectamine 3000 (Invitrogen, Grand Island, NY, USA) according to the manufacturer’s procedure. After 48 hours, cells were used for migration assay or expression analysis. The siRNA sequences were as listed in Table 1.

**Table 1:** Sequences of small interference RNA

Name	Sequence
siRNA‐1	
Sense	5‐CCAUUCCUCAUCCGUCAAATT‐3
Antisense	5‐UUUGACGGAUGAGGAAUGGTT‐3
siRNA‐2	
Sense	5‐GCACCUACUAUGUCACUUUTT‐3
Antisense	5‐AAAGUGACAUAGUAGGUGCTT‐3
siRNA‐3	
Sense	5‐GAUAUGUUUACUAAAGGACAAGUCA‐3
Antisense	5‐UGACUUGUCCUUUAGUAAACAUAUCAU‐3

### 2.6 Real‐time polymerase chain reaction

Overall, total RNA from MSCs were extracted with TRIzol reagent (Life Technologies, Grand Island, NY, USA), and the RNA samples were converted into cDNA using an Applied Biosystems High‐Capacity cDNA Reverse transcription Kit (Life Technologies) according to the manufacturer’s instructions. Real‐time PCR was performed using a QuantiTect SYBR Green PCR kit (Qiagen, Valencia, CA, USA) on a StepOne™ Real‐Time PCR System (Applied Biosciences, Grand Island, NY, USA). The primers used in the PCR are described in Table 2.

**Table 2:** Sequences of primers for qPCR

Gene name	Primer sequence	Size of product
HIF‐1α		
Forward	5‐TGGTATTATTCAGCACGACTT‐3	324 bp
Reverse	5‐GGAGACATTGCCAGGTTTAT‐3	
SDF‐1		
Forward	5‐ACGGCTGAAGAACAACAACA‐3	263 bp
Reverse	5‐TATGCTATGGCGGAGTGTCT‐3	
CXCR4		
Forward	5‐GGGGACATCAGTCAGGG‐3	360 bp
Reverse	5‐GTGGAAGAAGGCGAGGC‐3	
β‐actin		
Forward	5‐ATCATGTTTGAGACCTTCAACA‐3	318 bp
Reverse	5‐CATCTCTTGCTCGAAGTCCA‐3	

### 2.7 Western blotting

The cells were washed with ice‐cold PBS, lysed in RIPA buffer with protease inhibitors (Thermo Fisher Scientific, Rockford, IL, USA) on a rotation wheel for 1 hour at 4°C. After centrifugation at 10 000 *g* for 10 minutes, the supernatant was collected as protein extract. Protein concentration was measured by BCA kit inhibitors (Thermo Fisher Scientific, Rockford). Equal concentrations of proteins were fractionated by electrophoresis on 8% or 10% acrylamide gels and were transferred onto a polyvinylidene fluoride membrane (Millipore, Billerica, MA, USA) membrane, followed by blotting with antibodies against HIF‐1α (cat no. ab1; AbCam, Cambridge, MA, USA), SDF‐1 (cat no. ab157772; Abcam), CXCR4 (cat no. ab1670; Abcam) and β‐actin (cat no. ab8226; Abcam) followed by secondary staining with horseradish peroxidase‐conjugated immunoglobulin G. Protein expression was detected using an Image Reader (LAS‐3000 Imaging System; Fuji Photo Film, Tokyo, Japan).

### 2.8 Statistical analysis

All data are expressed as the mean ± SEM from at least three independent experiments. The differences between two groups were analysed with the two‐tailed unpaired Student’s *t* test, and differences between multiple groups were analysed with one‐way ANOVA followed by Tukey’s test, using Prism GraphPad (La Jolla, CA, USA). A value of *P* < 0.05 was considered statistically significant.

## 3 RESULTS

### 3.1 CD markers and cell morphology of mouse mesenchymal stem cells

**Figure 1:**
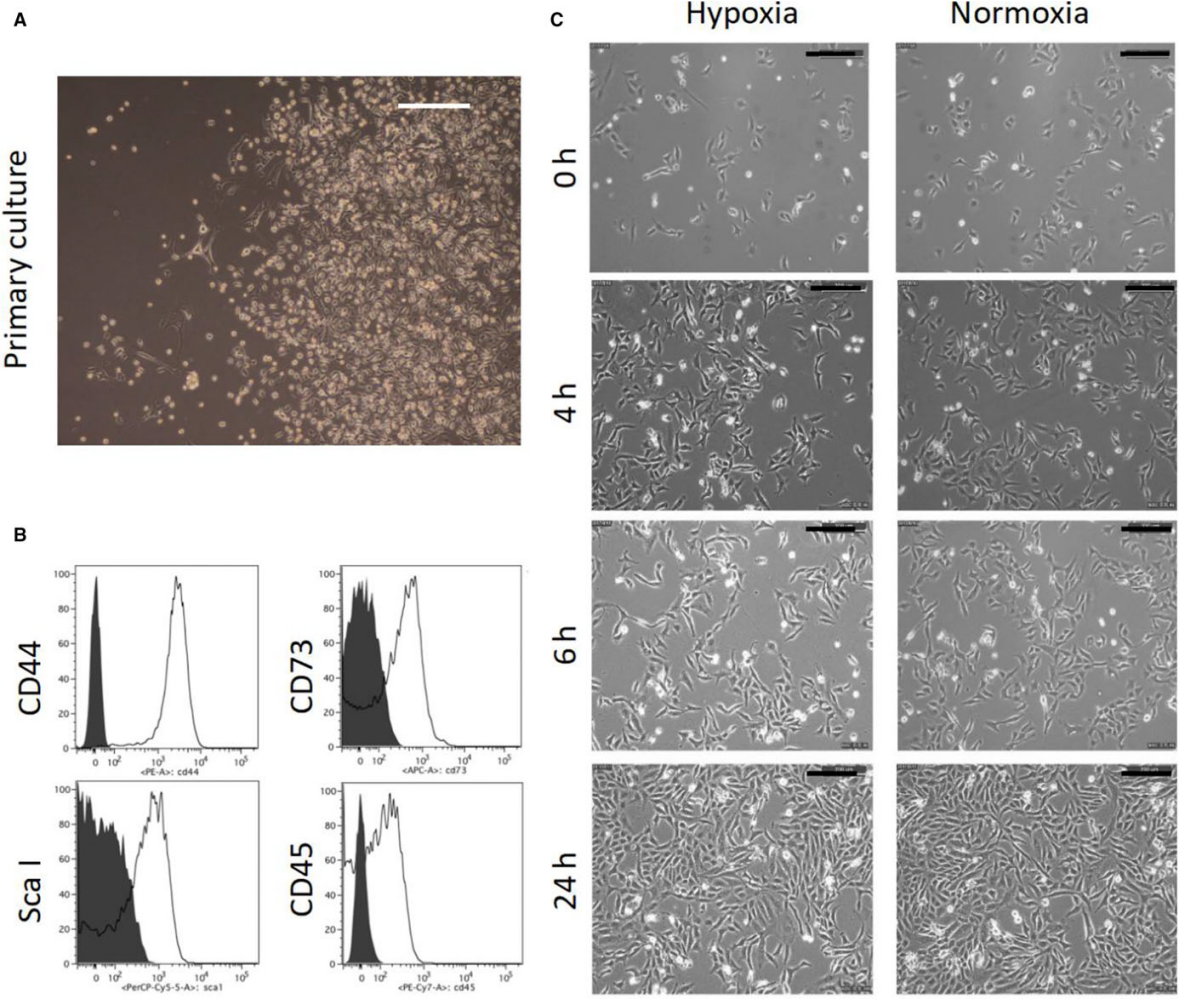
Characterization of murine MSCs. (A) Morphology of primary MSCs isolated from mouse bone marrow. MSCs easily form large colonies. (B) Histogram of flow cytometry for cell surface marker of MSCs. (C) Morphology of MSCs under normoxia and hypoxia conditions. Bar equals 200 µm in all panels

BM‐MSCs were isolated by their adhesion to cell culture surfaces and cultured in normoxic incubator (21% oxygen) for the first few passages. In primary culture of MSCs, they form large colonies on plastic surface (Figure 1A). Flow cytometric analysis was performed on cells cultured in normoxia with markers commonly used to characterize mouse MSCs, namely, CD44, Sca‐I, CD73 and CD45.^17^ The results of flow cytometry indicated that of mouse MSCs were positive for CD44, CD73 and Scal‐I (Figure 1B). A small portion of our MSCs were expressing CD45. This indicates the contamination of haematopoietic cells in MSCs at earlier passages. Increased cell passage number could result in a significant reduction of these contaminating cells. At P6, cells cultured in normoxia consisted of a heterogeneous cell population including convex round, convex spindled and flattened spindled morphology. The cellular morphology of BM‐MSCs in 1% oxygen was obviously more flattened spindle‐shaped and less convex compared with BM‐MSCs in 21% oxygen (Figure 1C).

### 3.2 Hypoxia increases MSC migration by enhancing SDF‐1/CXCR4 signalling

**Figure 2:**
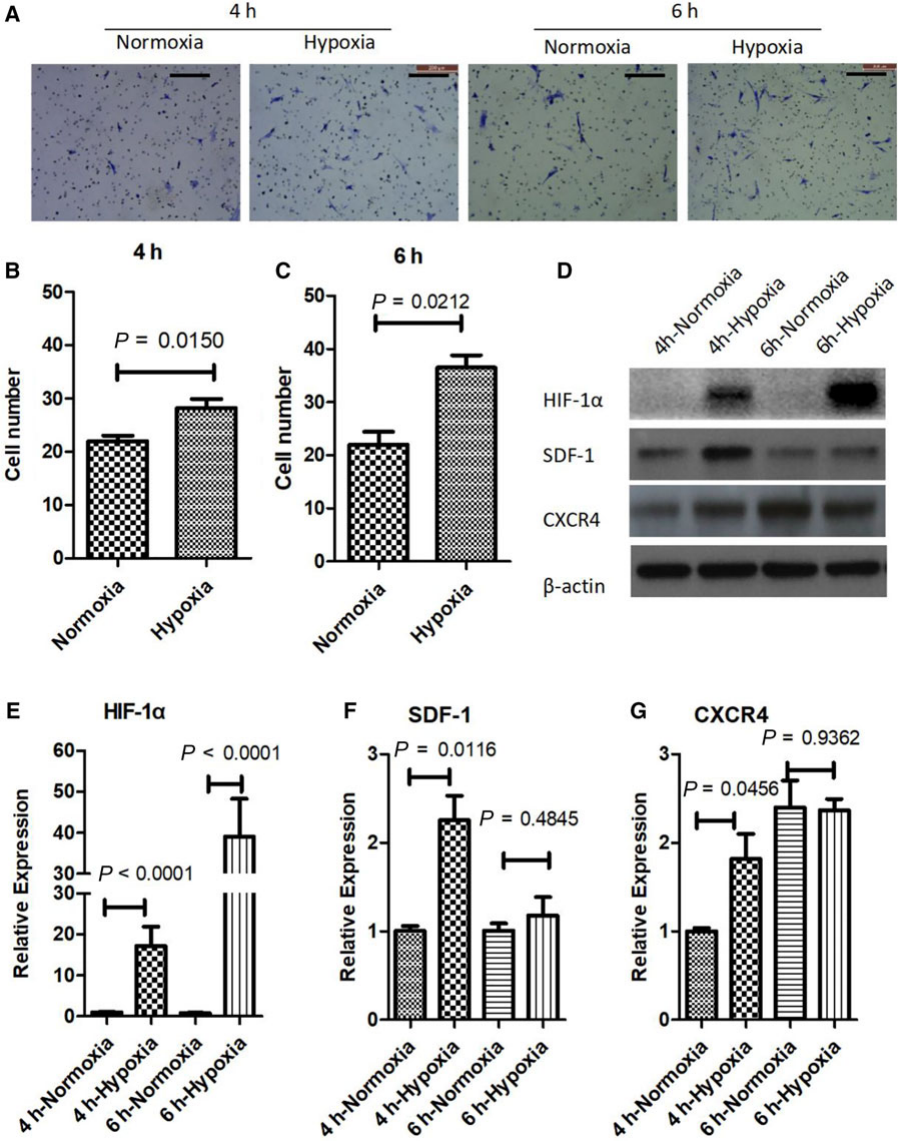
Hypoxia increases migration of MSCs. (A) Migrated cells were visualized by methylene blue staining, bar = 200 µm. (B, C) Numbers of migrated cells were counted. (D) Western blot was performed to reveal the expression of HIF‐1α, SDF‐1 and CXCR4 at protein level. (E‐G) qPCR was performed to examine the expression of HIF‐1α, SDF‐1 and CXCR4 at mRNA level. *P*‐values was calculated with Student’s *t* test in all panels

To validate the role of hypoxia in MSC chemotaxis migration, we performed transwell migration assays of MSCs at the presence of SDF‐1, under normoxia (21% oxygen) and hypoxia (1% oxygen). As shown in Figure 2A, many cells migrated to lower chamber as attracted by SDF‐1 at 4 hours in normoxia. However, more cells have been observed to migrate to the lower chamber in hypoxia at the same time. This impression of difference was further enhanced at 6 hours in culture. Quantification of the numbers of migrated cells was performed. Figure 2B,C shows the numbers of migrated cells with statistical results. At both 4 and 6 hours, there are more cells migrated in hypoxia than in normoxia. To further investigate the molecular mechanism of hypoxia‐induced MSC migration, we performed Western blot and qPCR to examine the expression level of HIF‐1α, SDF‐1 and CXCR4. In line with literature, the protein of HIF‐1α was only detected in hypoxic condition (Figure 2D). SDF‐1 expression was highly promoted by hypoxia at 4 hours after treatment, but not at 6 hours. Regarding, CXCR4 expression, there was only slight increase in hypoxia, compared to normoxia. The results of qPCR basically confirmed our impression about gene expression changes (Figure 2E‐G).

### 3.3 Small interference RNA successfully knock‐down the expression of HIF‐1α

**Figure 3:**
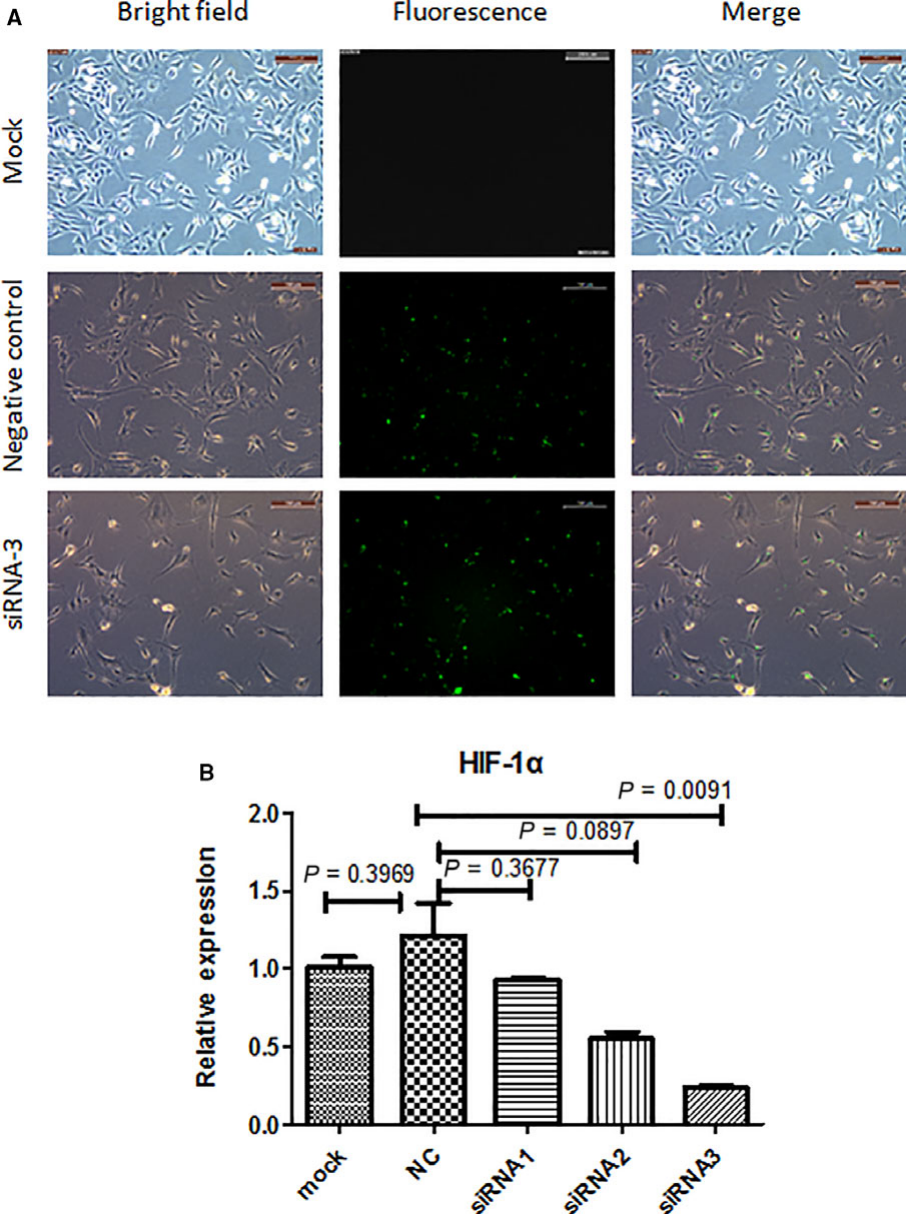
Transfection and knock‐down efficiency of small interference RNAs. (A) Green fluorescence indicates the efficiency of transfection, bar = 200 µm. (B) qPCR results indicate the knock‐down efficiency of different small interference RNAs. *P*‐values were calculated with one‐way ANOVA followed by Tukey’s test

In investigate the role of HIF‐1α in hypoxia‐induced chemotaxis migration, we designed three pairs of small inferences RNA to knock down the expression of HIF‐1α. Fluorescent images indicated the transfection efficacy of siRNA (Figure 3A). siRNA‐3 had a similar transfection efficiency as negative control. Results of qPCR indicated that siRNA‐3 could reduce the expression of HIF‐1α to the lowest level (Figure 3B). In the following experiments, only siRNA‐3 was used for knocking down of HIF‐1α.

### 3.4 Knocking down HIF‐1α abolished hypoxia induced chemotaxis migration in MSCs

**Figure 4:**
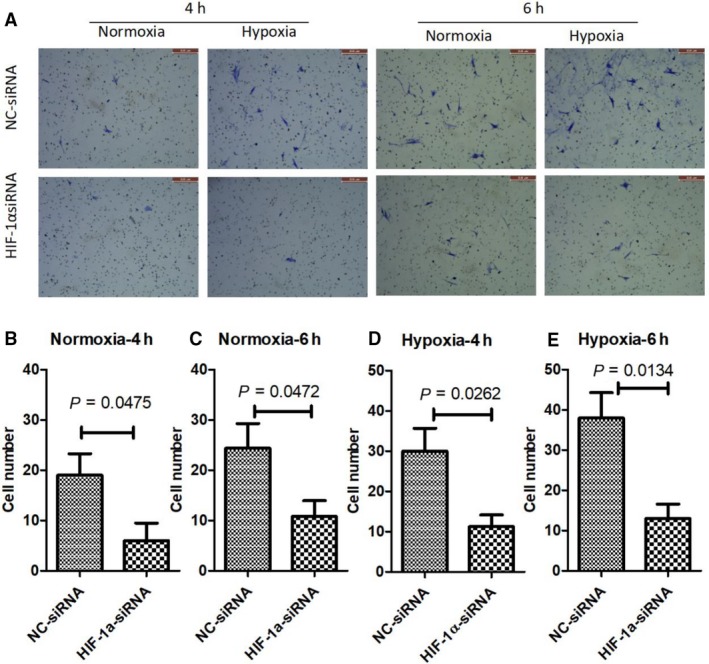
Knocking down of HIF‐1α reduces MSC migration. (A) Migrated cells were visualized by methylene blue staining, bar = 200 µm. (B‐E) Numbers of migrated cells were counted. *P*‐values was calculated with Student’s *t* test in all panels

It has been reported that Hif‐1α controlled chemotaxis towards the chemokine SDF‐1 by regulating expression of its receptor CXCR4.^18^ To demonstrate the function of Hif‐1α in the chemotaxis migration of MSCs, we knock down the expression of Hif‐1α by small interference RNA and examined the migration of MSCs by transwell device. Methylene blue staining revealed the cells that pass through the membrane. Less cells were observed in HIF‐1α knock down group, comparing to negative control group, in both normoxia and hypoxia, at 4 and 6 hours (Figure 4A). Quantification of the cell numbers showed that differences in cell numbers in all conditions are statistically significant (Figure 4B‐E).

### 3.5 Knocking down HIF‐1α reduces activation of SDF‐1/CXCR4 signalling axis in MSCs

**Figure 5:**
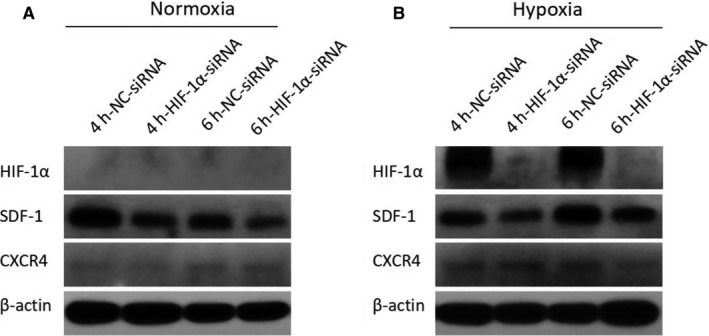
Knocking‐down of HIF‐1α surprises SDF‐1 expression in MSCs at the protein level. Western blot was performed to reveal the expression of HIF‐1α, SDF‐1, and CXCR4 at the protein level in either normoxia (A) or hypoxia (B). Hours of treatment was indicated in figure captions. NC=non‐specific control

**Figure 6:**
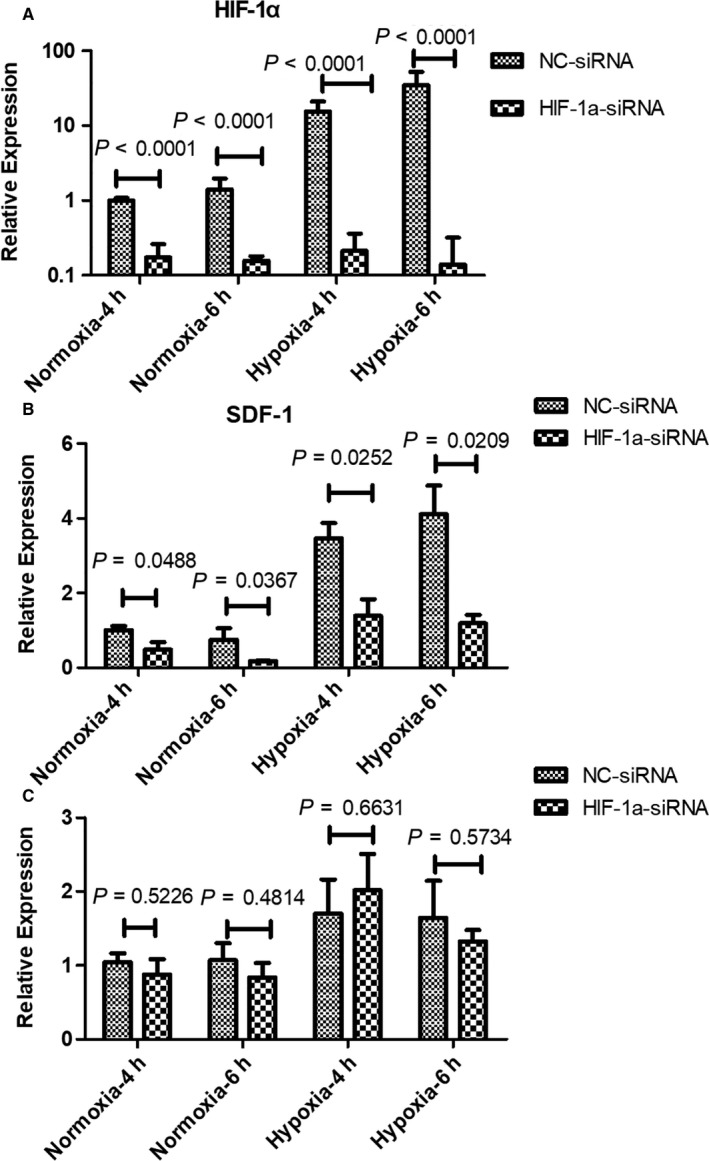
Knocking‐down of HIF‐1α surprises SDF‐1 expression in MSCs at mRNA level. qPCR was performed to examine the expression of HIF‐1α (A), SDF‐1 (B) and CXCR4 (C) at mRNA level. *P*‐values were calculated with Students t‐test in all panels. Hours of treatment was indicated in figure captions. NC=non‐specific control

As shown in Figure 5A, we cannot detect any expression of HIF‐1α in normoxic condition by Western blot. SDF‐1 expression was slightly reduced by HIF‐1α knocking down, meanwhile, the expression of CXCR4 was shown at low level, and not changed too much. Under hypoxic condition, HIF‐1α knocking down almost abolished the expression of HIF‐1α completely in MSCs. Interestingly, there is an obvious reduction of SDF‐1 expression with knocking down of HIF‐1α (Figure 5B). Again, there was litter variation on CXCR4 expression. Next, we performed qPCR to confirm the expression profiles of SDF‐1 and CXCR4 at mRNA level. Only under hypoxic condition, HIF‐1α knocking down suppressed SDF‐1 expression (Figure 6B). CXCR4 expression seemed not be regulated by HIF‐1α (Figure 6C).

## 4 DISCUSSION

It is previously reported that infusion of MSC overexpressing HIF‐1α promotes myocardial healing in an experimental rat model of myocardial infarction.^19^ As described in previous studies, signalling pathways related to several paracrine factors and interleukins are up‐regulated in HIF‐MSCs.^20^ In this study, we reported that hypoxia increased chemotaxis migration of MSCs. Using small interference RNA, we knock down the expression of HIF‐1α in MSCs. Combining the results of migration assay and expression at RNA and protein level, we demonstrated a novel mechanism that control the increase of MSC migration. This mechanism involved HIF‐1α mediated SDF‐1 expression.

Stem cell therapy using MSCs has been explored for the treatment of various degenerative diseases. However, the mechanisms of action are poorly understood. MSC migration is a critical factor determining the efficacy of stem cell therapy because the therapeutic effect of MSCs can only be expected after the proper engraftment of transplanted MSCs to the damaged tissues.^21^ Ceradini and colleagues reported that the recruitment of progenitor cells into the regenerating tissues was regulated by hypoxic gradients via the HIF‐1 induction of SDF‐1, which binds to CXCR4 on circulating progenitor cells.^22^ In a previous clinical study, it was demonstrated that MSC co‐infusion improved haematopoietic stem cell engraftment through restoration of a normal level of SDF‐1 in eight patients with acute myeloid leukaemia undergoing haematopoietic stem cell transplantation.^23^ However, the interactions between HIF‐1α and the signalling molecules, such as integrin, MMPs and Rho GTPases, under hypoxia and their influences on MSC migration have not been fully elucidated.^24^ HIF‐1α is a pivotal transcription factor regulating the adaptive response to hypoxia^25^ and numerous proteins interact directly with HIF‐1 to enhance or reduce its function.^26^

Our results further suggested that HIF‐1α expression is necessary for hypoxia induced MSC migration. Previous work has shown that cytokine SDF‐1 and its receptor CXCR4 are able to regulate cell migration.^27^ And it has been reported that increased SDF‐1 expression is responsible for the enhanced migration of MSCs.^28^ Our results linked the up‐regulation of SDF‐1/CXCR4 signalling with hypoxia induced HIF‐1α expression. This data help us to put together all pieces of a puzzle for the mechanism which explained why hypoxia could induce MSCs migration. When cultured in a hypoxic condition, MSCs first boosted the expression of HIF‐1α. Then, HIF‐1α alone or together with some other regulatory genes up‐regulated the expression of SDF‐1. SDF‐1 sub‐sequentially enhanced the migration of MSCs.

Taken together, our results suggest that hypoxia‐induced expression of HIF‐1α plays a critical role for the migratory effects of MSCs. The mechanism involves up‐regulation of the SDF‐1/CXCR4 signalling pathways that lead to enhanced MSC mobility and migration.

## 5 CONCLUSION

Hypoxia culture condition significantly improves the migratory effects of MSCs. Furthermore, the molecular mechanism of this procedure has been suggested to be controlled through HIF‐1α mediated SDF‐1 expression. Further experiments using in vivo model may be helpful to validate the migratory effects of MSCs under hypoxia, however, we believe that MSCs play an important role in wound healing by expressing high level of HIF‐1α.

## CONFLICT OF INTEREST

All the authors confirm that they have no competing interests.

## AUTHOR CONTRIBUTION

WX, RX and YW designed the experiments; WX, RX and YW performed the experiments; WX and RX analysed the data; WX and YW drafted the manuscript. All authors discussed the results and commented on the manuscript.
